# Fractionation of the Caspian sand goby epidermal exudates using membrane ultrafiltration and reversed-phase chromatography: an investigation on bioactivities

**DOI:** 10.1038/s41598-024-52126-z

**Published:** 2024-01-19

**Authors:** Mohammad Akhavan-Bahabadi, Hamed Paknejad, Aliakbar Hedayati, Mehran Habibi-Rezaei

**Affiliations:** 1 Department of Fisheries, Faculty of Fisheries and Environmental Sciences, Gorgan University of Agricultural Sciences and Natural Resources, Gorgān, Iran; 2 Protein Biotechnology Research Lab (PBRL), School of Biology, College of Science, University of Tehran, Tehran, Iran; 3 Present Address: National Research Center of Saline Water Aquatics, Iranian Fisheries Science Research Institute, Agricultural Research, Education and Extension Organization (AREEO), Bafq, Yazd Iran

**Keywords:** Biotechnology, Drug discovery, Immunology, Microbiology, Diseases, Medical research

## Abstract

Bioactive peptide-based drugs have gained exceeding attention as promising treatments for infectious and oxidative-stress-related diseases, are exacerbated by the advent and spread of various multidrug-resistant bacteria and industrial lifestyles. Fish skin mucus has been recognized as a potential source of bioactive peptides, providing the first line of fish defense against invading pathogens which are targeted here to be explored as a new source of biopharmaceutics. Peptide fractions were isolated from the epidermal exudates of Caspian sand goby, *Neogobius fluviatilis pallasi,* by solid-phase extraction (SPE), ultrafiltration, and reversed-phase chromatography. The resulting fractions were characterized for their antibacterial and antioxidant properties, and results showed that the molecular weight fraction < 5 kDa represented the highest (*p* < 0.05) bacterial inhibition activity against *Staphylococcus aureus* and *Bacillus subtilis* as well as scavenging activity against DPPH and ABTS radicals. Overall, these results introduce the epidermal mucus of Caspian sand goby as a valuable source of bioactive compounds that can be considered new and efficient biopharmaceutics.

## Introduction

In recent years, the advent and spread of diverse multidrug-resistant bacteria and oxidative stress-related diseases have become a serious concern not only to the medical field but also to public health. It has been considerably hastened by human activities, including immoderate and improper antibiotic application in clinical practice, livestock, and the aquaculture industry, as it is the fastest-growing part of agriculture^1^. Besides, it is predicted that worldwide death owing to antibiotic resistance will reach 10 million by 2050^2^. Consequently, it is crucial to explore and develop alternative antimicrobial agents that will not develop resistance as an urgent action. Bioactive peptides (BAPs) are found in a wide variety of sources, which are primarily inactive within the specific molecular structure of parent proteins and can be released during food processing or enzymatic digestion^3^. These peptides are mainly cationic, low-molecular-weight, and hydrophobic and exhibit a wide range of bioactivities, including immunomodulatory, antihypertensive, anticancer, antioxidant, antiviral, wound healing, and antimicrobial activities, including multidrug-resistant bacteria, which last activity brings them to be known as antimicrobial peptides (AMPs)^4^.

Antioxidants play an essential role in human health, protecting the body against reactive oxygen species (ROSs) and reactive nitrogen species (NOSs). These species are reactive due to unpaired valence shell electrons with short life spans^5,6^, which make them active against lipids, carbohydrates, proteins, and nucleic acids especially causing lipid peroxidation and cell membrane damage in the cell^5^. They comprise a range of hydroxyl radical (HO^·−^), superoxide anion (O_2_^·−^), peroxyl radical (RO^·−^), and non-free radical species like hydrogen peroxide (H_2_O_2_), nitric oxide radical (NO^·^) as well as hypochlorous acid (HOCl). Under normal conditions, the generation and elimination of ROSs can be efficiently balanced to make them present at low and steady stationary levels to play essential roles in cell signaling and homeostasis in normal cells^7^. Managing redox status is crucial for cell viability, activation, propagation, and function of organs. They are generated as natural by-products of the normal aerobic metabolism of oxygen and are eliminated through combined enzymatic and non-enzymatic antioxidant approaches. Under pathological imbalanced conditions, they initiate the onset of various lifestyle disorders collectively known as oxidative stress-related diseases, including type 2 diabetes mellitus (T2DM), Alzheimer’s disease (AD), cardiovascular diseases (CVD), cancer, etc^5,6^. As a result, they are dual edge sward whether they act as damaging or defending factors depending on the balance between ROS production and disposal at the right time and place. Furthermore, lipid oxidation in food processing and storage results in degeneration, creating unpleasant and annoying odors and tastes that lower quality and nutritional values^8^. This makes serious attention to antioxidants as food additives to prevent lipid peroxidation and its harmful complications besides the lower food quality. Synthetic antioxidants such as butylated hydroxytoluene (BHT), butylated hydroxyanisole (BHA), sodium benzoate, and potassium sorbate are economical and practical but have been reported with possible health risks most importantly, their amyloidogenic effects^9,10^, liver damage or cancers^11^ which are collectively preferred natural rather than synthetic preservatives^12^.

Gobiidae is the most varied fish family in the Caspian Sea, and the *Neogobius* genera have been one of the most numerous and widely spread Caspian Sea gobies with great ecological importance as well as playing a considerable role in its food chain^13^. *Neogobius fluviatilis pallasi *skin is also characterized by an increased variety of pathogen burdens^14^. The cutaneous mucus layer is considered the critical component of the non-specific immune system, which is secreted via mucous glands or goblet cells, as well as includes bioactive compounds such as AMPs^15^ which their expression is begun in response to cytokines or pathogen-associated molecular patterns (PAMPs), and damage-associated molecular pattern (DAMP) such as Toll-like receptors (TLRs) presence in mucus^16^.

To date, fish with over 144 AMPs, are a great source of these peptides, as they express all of the major classes of AMPs, including defensins, cathelicidins, hepcidins, histone-derived peptides, and a fish-specific class, namely piscidins^4^. Two later classes have been found in the fish epidermal mucus. Piscidin family members such as pardaxin, pleurocidins, Myxinidin, and grammistin are chiefly the main ingredients of the fish skin mucus and represent robust biological activities to a wide range of Gram-positive and Gram-negative bacteria, particularly versus drug-resistant ones. Besides, piscidins have antiparasitic, antifungal, antiviral, anti-tumor, and wound healing activities. It also has been proven that piscidins can accompany other AMPs to heighten the efficiency of their activity^4,17^.

Although it is possible to isolate the BAPs from different origins, their applications are restricted by the intrinsic issues related to natural peptides including, low bioavailability, short half-life, cytotoxicity, degradation by enzymes, salt sensitivity, poor membrane permeability, rapid clearance, etc. On the other hand, many strategies have been investigated to circumvent these problems and to improve the efficacy of BAPs. These include chemical modification of peptides and the use of delivery vehicles^18^.

Many sources, including amphibians and mammalians, have been studied to discover mucosal AMPs. Also, the by-product hydrolysates (FPH), collagen, and gelatin-derived-antioxidant peptides from various marine aquatic parts have been identified^12,19–22^; however, a few AMPs (Table 1) and no antioxidant peptides from fish skin mucus have been reported. Here, we are reporting the antimicrobial and antioxidant activities of the epidermal exudates fractionations of *N. fluviatilis pallasi*.

**Table 1:** Fish skin exudates-isolated AMPs.

Protein/peptide	Fragment	Source	Activity	Reference
Pardaxins				
Pa1		*Pardachirus pavoninus*	H	^23^
Pa2		*P. pavoninus*	H	^23^
Pa3		*P. pavoninus*	H	^23^
Pa4		*Pardachirus marmoratus*	G+ /G−/F/V/C/W/H	^24^
Pa5		*P. marmoratus*	H	^25^
Pleurocidins				
WF1		*Pseudopleuronects americanus*	G+/G/F/C/B	^26^
WF2		*P. americanus*	G+/G−/F	^27^
WF1L		*P. americanus*	G+/G−/F	^27^
WFX		*P. americanus*	G+/G−/F	^27^
WFY		*P. americanus*	G+/G−/F	^27^
WF3		*P. americanus*	G+/G−/F	^28^
WF4		*P. americanus*	G+/G−/F	^28^
Grammistins				
Grammistin Pp2b		*Pogonoperca punctata*	G+/G−/H	^29^
Grammistin Pp3		*P. punctata*	G + / G-/ H	^29^
Grammistin Pp1		*P. punctata*	G+/G−/H	^29^
Grammistin GsG		*Grammistes sexlineatus*	G+/G−/H	^29^
Grammistin GsF		*G. sexlineatus*	G+/G−/H	^29^
Grammistin GsC		*G. sexlineatus*	G+/G−	^29^
Grammistin GsA		*G. sexlineatus*	G+/G−	^29^
Grammistin GsB		*G. sexlineatus*	G+/G−/H	^29^
Myxinidin		*Myxine glutinosa*	G+/G−/F/B/W	^30^
Pelteobagrin		*Pelteobagrus fulvidraco*	G+/G−/F	^31^
AJN-10		*Anguilla japonica*	G+/G−/F	^32^
H1 (Nucleus)	Whole protein (20.7 kDa)	*Salmo salar*	G−	^33^
H1 (Nucleus)	SAMP H1	*S. salar*	G+/G−	^34^
H1 (Nucleus)	N-terminus (HSDF-1)	*Onchorhynchus kisutch*		^35^
H1 (Nucleus)	C-terminus (oncorhyncin II)	*O. mykis*		^36^
H1 (Nucleus)	Oncorhyncin-I	*O. mykis*		^36^
Histone H2A (Nucleus)	Whole protein (13.5 kDa)	*Ictalurus punctatus*	G+/G−/F	^37^
Histone H2A (Nucleus)	Whole protein (13.5 kDa)	*O. mykiss*	G+/H	^38^
Histone H2A (Nucleus)	N-terminus (hipposin)	*Hippoglossus hippoglossus*	G+ /G−	^39^
Histone H2A (Nucleus)	N-terminus (parasin-1)	*Parasilurus asetus*	G+/G−/F	^40^
Histone H2B (Nucleus)	Whole protein (13.8 kDa)	*Gadus morhua*	G−	^41^
Histone H2B (Nucleus)	Whole protein (15.5 kDa)	*I. punctatus*	G−/F	^37^
Histone H3 (Nucleus)	~ 1 kDa	*Myxine glutinosa*		^42^
Protein H6	Whole protein (oncorhyncin III)	*O. mykiss*	G+, G−	^43^
40 Rsp (S30)	Whole protein (6.7 kDa)	*O. mykiss*	G+	^44^
60 Rsp (L40)	Whole protein (6.4 kDa)	*G. morhua*	G+, G−	^41^
60 Rsp (L36A)	Whole protein (12.3 kDa)	*G. morhua*	G+, G−	^41^
60 Rsp (L35)	Whole protein (14.2 kDa)	*G. morhua*	G+	^41^
Haemoglobinβ (Hgbβ)	PcfHb	*Potamotrygon cf. henlei*	G+, G−/F	^45^

G + , G-: Antibacterial activity against Gram positive (G +) and Gram negative (G-); F: Antifungal effect; V: Antiviral effect; C: Anticancer effect; B: Antibiofilm effect; W: wound healing effect; H: Hemolytic activity.

## Materials and methods

### Materials

Fish weighing 50 ± 5 g were collected from Gorgan Gulf, Ashoradeh. Human pathogens (*Escherichia coli* ATCC 27325 (American Type Culture Collection) (Gram-negative), *Staphylococcus aureus* ATCC 15752 (Gram-positive), *Pseudomonas aeruginosa* ATCC 27853 (Gram-negative), and *Bacillus subtilis* ATCC 62037 (Gram-positive) and fish pathogens (*Vibrio harveyi* PTCC 1755 (Persian Type Culture Collection) (Gram-negative) and *Yersinia ruckeri* PTCC 1888 (Gram-negative) were prepared as preserved strains from the Pasteur Institute of Iran. All strains were stored at − 20 °C until use. The centrifugal ultrafiltration device with molecular weight cut-offs (MWCO) of 3, 5, 10, and 30 kDa and the CHROMABOND solid phase extraction (SPE) columns for sample preparation were obtained from Sartorius Stedim Biotech (Germany) and MACHEREY-NAGEL (Germany), respectively. 1,1-diphenyl-2-picrylhydrazyl (DPPH˙), 2, 2′-azino-bis (-ethylbenzothiazoline-6-sulphonic acid) diammonium salt (ABTS), and all other chemicals used in the experiments were from Sigma–Aldrich (Missouri, USA).

### Epidermal mucus collection and peptide purification

Epidermal excretions were obtained from 60 to 70 fish, as reported previously^46^. In brief, 3–5 fish were transferred into a polyethylene bag having 10 mL sodium chloride 100 mM. The fish were gently moved forth and back inside the bag for 1–2 min to collect the skin mucus. Then, fish were returned to the sea. The purification of peptides was performed based on Park et al.^40^ method with minor modifications^40^. The skin-secreted mucus was homogenized by a Waring blender (Eberhard Bauer D-7300 Esslingen) in the chilled extraction buffer 0.2 M sodium acetate buffer pH 7.4 including 0.2% Triton X-100, and 1 mM phenylmethylsulfonyl fluoride (PMDF), at 1:4 (v/v). The homogenate was stirred using a magnetic stirrer (Heidolph MR 3001 k) for 24 h at 4 °C, then was centrifuged at 20,000×*g* for 30 min at 4 °C using Sigma 2-16 KL. The supernatant was subjected to SPE cartridges Sep-Pak C18 which were activated by 80% acetonitrile (ACN), including 0.1% (v/v) trifluoroacetic acid (TFA) as buffer A and flushed with 0.1% (v/v) TFA as buffer B to eliminate the excess ACN. Sep-Pak C18 cartridges were loaded with the supernatant and washed with 20 ml of buffer B; then, the trapped peptides were eluted with 6 ml of buffer A. The eluates were freeze-dried and then resolved in buffer B, stored at − 70 °C until use. The eluates from each cartridge were pooled and were sequentially passed through ultrafiltration membranes with molecular weight cut-offs of 3, 5, 10, and 30 kDa in the stirred ultrafiltration cells based on manufacturer instructions (6000×*g*, 10 min, 4 °C), and the obtained fractions freeze-dried and stored at − 20 °C until analysis.

### Reverse-phase high-performance liquid chromatography (RP-HPLC)

The bioactive fraction was applied to an 8 mm × 250 mm RP-HPLC column (CECIL, Italy) equilibrated with 0.1% (v/v) trifluoroacetic acid (TFA)/water. Elution was performed using 0.1% (v/v) TFA/water over 10 min, followed by a linear gradient of 0–80% acetonitrile in 0.1% (v/v) TFA over 70 min at a flow rate of 1 ml min^−1^, and final elution with 80% acetonitrile in 0.1% (v/v) TFA. The eluate was monitored at 214 nm, and UV-absorbing peaks were collected, freeze-dried, resuspended in water, and assayed for bioactivities. The fractions with antimicrobial activity were pooled and further purified by C18 RP-HPLC under modified conditions (with a gradient of 50–80% (v/v) acetonitrile in 0.1% (v/v) TFA) and then concentrated as before.

### Protein assay and electrophoresis

The total protein content of the skin mucus and the fractions were estimated using the Bradford^47^ method, in which bovine serum albumin (BSA) was used as a standard. The samples were investigated to determine the purity and the molecular weight profile using Tricine SDS-PAGE, according to Schägger^48^. Unless otherwise, 4% acrylamide stacking gel and 15% acrylamide separating gel was adopted. The protein marker (polypeptide, Cinnagen) was used, and Coomassie brilliant blue staining was performed.

### Antimicrobial assay

The antimicrobial activities of the crude extract of epidermal mucus, SPE, ultrafiltration, and RP-HPLC-purified fractions were studied for activity towards a range of pathogenic Gram-positive and Gram-negative bacteria (2.1. Materials) by the radial diffusion assay (RDA). The minimal inhibitory concentration (MIC) and the minimum bactericidal concentration (MBC) were achieved following the microtitre broth dilution method, as described previously^46^. Antibacterial activity of the purified fraction was assessed by radial diffusion assay as above, following digestion with a final concentration of 60 µg ml^−1^ proteinase K for 60 min at 37 °C.

### Antioxidant assay

ABTS free radical scavenging assay was accomplished according to^49^. The assay reagent was prepared by dissolving 0.5 g manganese oxide as an oxidizing agent in 10 ml solution of 2, 2-azinobis (3-ethylbenzothiazoline-6-sulfonic acid) diammonium salt radical (ABTS) at 5 mM in H_2_O which was irregularly shaken at room temperature for 20 min. Excess MnO_2_ was first eliminated by centrifuge (5000×*g*, 20 min), then by filtering using a 0.2 µM syringe-end filter. The ABTS solution was diluted with 5 mM phosphate buffer saline (PBS), pH 7.4, to absorb of 0.7 (± 0.02) at 734 nm. Trolox was used as standard at the final concentration range of 0–200 µM. 70 µL of samples or Trolox standards were added to 700 µL of ABTS solution, vortexed for 10 s, incubated for 300 s, and absorbances were read out trice by spectrophotometer (UV–Vis, Jenway) in which PBS was used as the blank. All steps were performed in the dark, at room temperature. The antioxidant activities were calculated as the mean value of the Trolox equivalent antioxidant capacity. The radical scavenging activities were also estimated using DPPH free radical scavenging assay according to the method by^49^ with minor modifications. In brief, 20 µL of samples were added to 250 µL of 0.1 mM—2, 2- diphenyl-1-picrylhydrazyl (DPPH) in ethanol and 750 µL of 80% ethanol, followed by the absorbance read out at 512 nm. Trolox was used as standard at the final concentration range of 0–200 µM. Measurements were done three times per sample. The antioxidant activities were calculated as the mean value of the Trolox equivalent antioxidant capacity. All steps were done trice and performed under room temperature, in the dark.

### Statistical analyses

All experiments were done in triplicate. The data was expressed as means with standard deviations. The one-way analysis of variance (ANOVA) was applied to compare and analyze the average of each treatment, and the Duncan’s multiple range test was used to analyze the significant differences in different groups (*p* < 0.05) (SPSS 17.0 software).

### Ethics approval

All experimental protocols were reported in accordance with ARRIVE guidelines and were approved by the Ethics Committee at Gorgan University of Agricultural Sciences and Natural Resources (Approval No. 9421024101, 2020/09/15). Also, all methods were carried out under the relevant guidelines and regulations.

### Equipment and settings

All the images were taken by a cell phone (Samsung Galaxy S6) and the paint software was used for image editing and dropping.

## Results

### Bioassay-guided fractionation

To identify the active component(s) responsible for the bioactivities, the epidermal mucus was extracted, concentrated, and partially purified by Sep-Pak C18 cartridges, followed by fractionation using the centrifugal filter devices with 4 different MWCOs (30, 10, 5, and 3 kDa) as well as HPLC technique.

**Figure 1:**
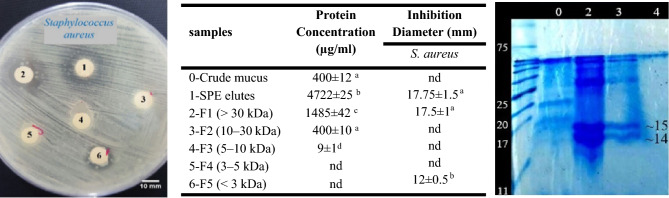
The table shows the numbered protein content and antibacterial activity of the crude epidermal mucus, SPE elutes, and fractions (F) F1 (˃ 30 kDa), F2 (10–30 kDa), F3 (5–10 kDa), F4 (3–5 kDa), and F5 (< 3 kDa) against *S. aureus*, (n = 3). Different letters indicate significant differences (*p* < 0.05). The culture plate at the left shows the antibacterial activity of SPE elutes and the fractions against *S. aureus*. The cropped SDS-PAGE of the samples has shown at right. The original image of full-length (uncropped) gel is presented in the Supplementary Fig. S1. It has been cropped owing to improve the clarity and conciseness of the presentation. Numbering has defined in the table. nd: Not detected.

Antimicrobial activity was not detected when skin mucus was applied directly to the radial diffusion assay; however, the activity was first detected after the mucus had been homogenized in the extraction medium, centrifuged, and the peptides in the supernatant were concentrated by SPE on a Sep-Pak C18 column (Fig. 1). This approach led to an increase of more than 10 times in skin mucus protein content and the observation of an antibacterial effect (Fig. 1).

The ultrafiltration of the SPE elutes resulted in fractions including F1 (˃ 30 kDa), F2 (10–30 kDa), F3 (5–10 kDa), F4 (3–5 kDa), and F5 (< 3 kDa). The fractions were individually collected and assayed for antimicrobial activity against two and four nominated Gram-positive and Gram-negative bacteria respectively, as mentioned in 2.1. Materials. Among nominated bacteria, only *S. aureus* represented sensitivity to SPE elute, F1, and F5 fractions (Fig. 1). The MW distribution patterns of the fractions were validated using Tricine SDS-PAGE analyses, as depicted in the Fig. 1. The protein content of different fractions showed that significant amounts of protein are missed in the fractionation process (Fig. 1); thereby, it was decided to use a filter (5-kDa). Therefore, antibacterial activity against bacterial species for fractions ˂ and ˃ 5-kDa took place after concentration with SPE and fractionation with filter 5-kDa.

**Figure 2:**
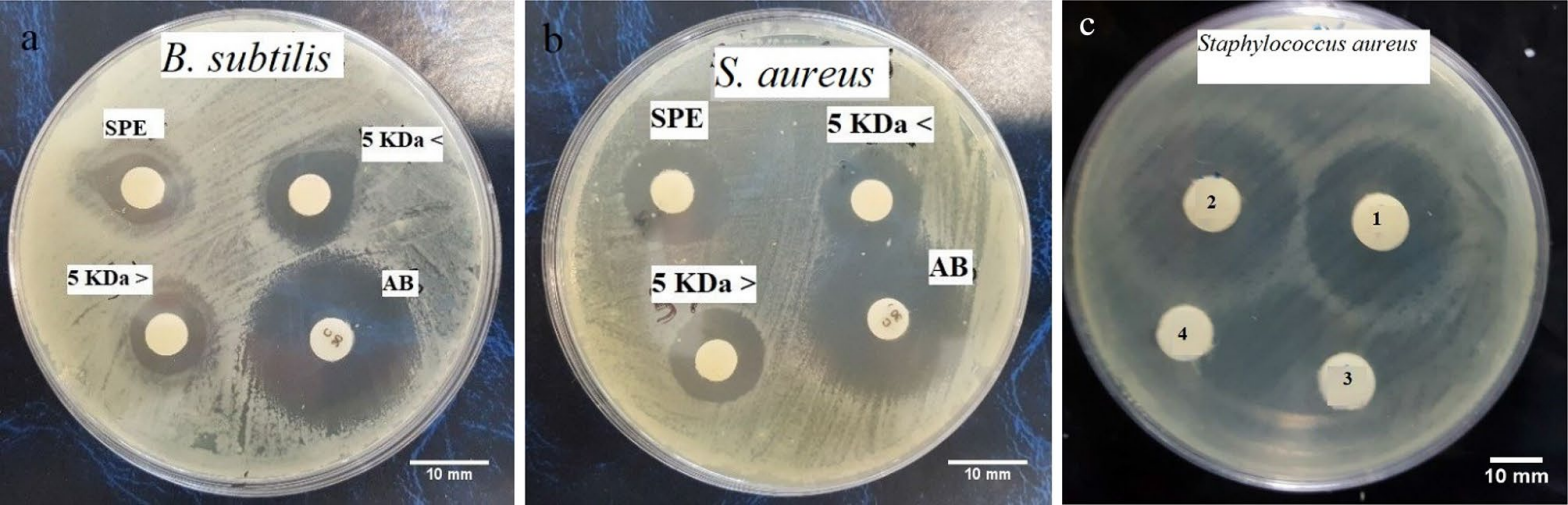
The result of Culture plates depicts radial diffusion assay (RDA) for the antibacterial activity of SPE elutes, and the fractions < 5 kDa, 5 kDa < , and blank sterile Antibiotic disk (AB) against *B. subtilis* (**a**) and *S. aureus* (**b**). The protein nature of both fractions ˂5 kDa, 5 kDa < has been confirmed by comparison of RDA for 5 kDa < and < 5 kDa without (1, 2) and with (3,4) digestion with proteinase K, respectively (**c**). * The amount of antibacterial activity of the fraction relation to the antibiotic activity (%). The entire result of MIC and MBC tests can be found as Supplementary Table S1, Supplementary Figs. S3 and S4.

The antibacterial activity investigation of ˂5 kDa and 5 kDa < fractions revealed the relative more potent antibacterial activity of < 5 kDa fraction against Gram-positive species, *S. aureus* and *B. subtilis* (Table 2, Fig. 2a,b). Nevertheless, there was no antimicrobial activity against the next four Gram-negative bacteria (Supplementary Fig. S2). The active substance appeared to be a peptide since it was inactivated entirely upon treatment with proteinase K (Fig. 2c).

**Table 2:** Protein content and antibacterial activity of the fractions ˂5 kDa, 5 kDa < and blank sterile antibiotic disk (AB) against *S. aureus* and *B. subtilis* (n = 3).

samples	Protein concentration (µg/ml)	Inhibition diameter (mm)		Percent value^a^ (%)		MIC (µg/ml)		MBC (µg/ml)	
		*S. aureus*	*B. subtilis*	*S. aureus*	*B. subtilis*	*S. aureus*	*B. subtilis*	*S. aureus*	*B. subtilis*
˃ 5-kDa	3265 ± 35	22.75 ± 2^a^	16.45 ± 1^a^	86.66 ± 2^a^	58.75 ± 1^a^	14.6	14.6	14.6	14.6
˂ 5-kDa	nd	19.25 ± 1^b^	12.25 ± 2^b^	73.33 ± 1^b^	43.75 ± 2^b^	8	8	17	17
AB	nd	26.25 ± 3^c^	28 ± 1.7^c^	100^c^	100^c^	nd	nd	nd	nd

Different letters indicate significant differences (*p* < 0.05). nd: Not detected.

In this study, the < 5 kDa peptide fraction exerted an effective antibacterial activity against *S. aureus* and *B. subtilis*, with MIC and MBC values of 14.6 µg/mL, while about the ˂ 5-kDa peptide fraction was 8 and 17 µg/mL respectively (Table 2). These findings indicated a considerable potential for the epidermal mucus-derived fractions of Caspian sand goby as a potent antibacterial agent.

### HPLC

**Figure 3:**
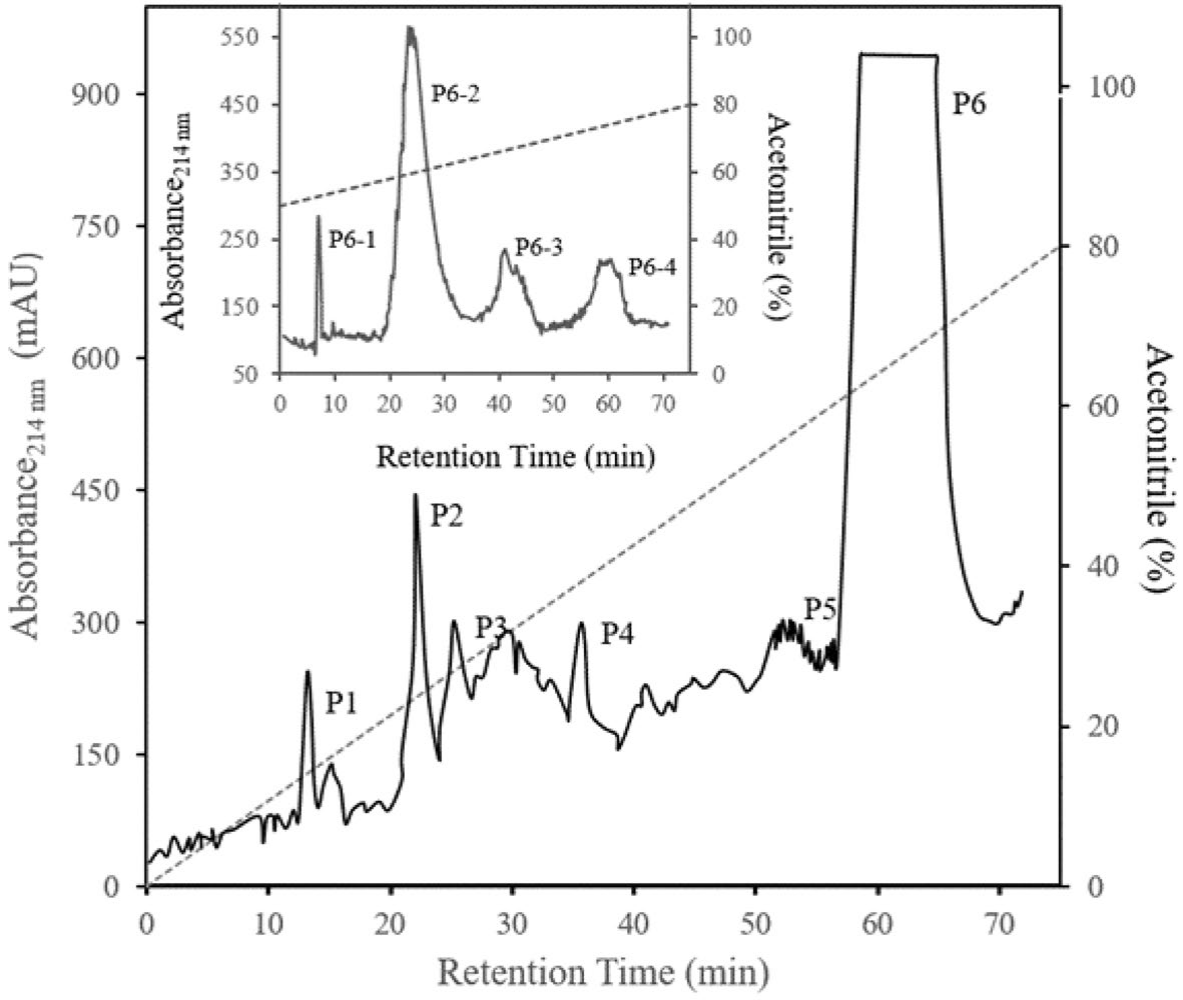
Reverse-phase HPLC analyses of < 5-kDa peptide fraction. The samples were loaded on an 8 mm × 250 mm RP-HPLC column (CECIL, Italy), and elutions were achieved with a linear gradient (dotted line) of acetonitrile in aqueous trifluoroacetic acid (80% acetonitrile/0.1% TFA). Absorbance was monitored at 214 nm (solid line). The inset represents the HPLC profile of peak 6 (P6) with a slow gradient (50–80% (v/v) acetonitrile containing 0.1% (v/v) TFA).

The highest relative activity rate against selected bacteria was exhibited by < 5 kDa peptide, which was further fractionated using semi-preparative C18 RP-HPLC. The fraction was first separated into six chromatographic peaks by preparative RP-HPLC (Fig. 3). This experiment was repeated several times with the same results.

**Figure 4:**
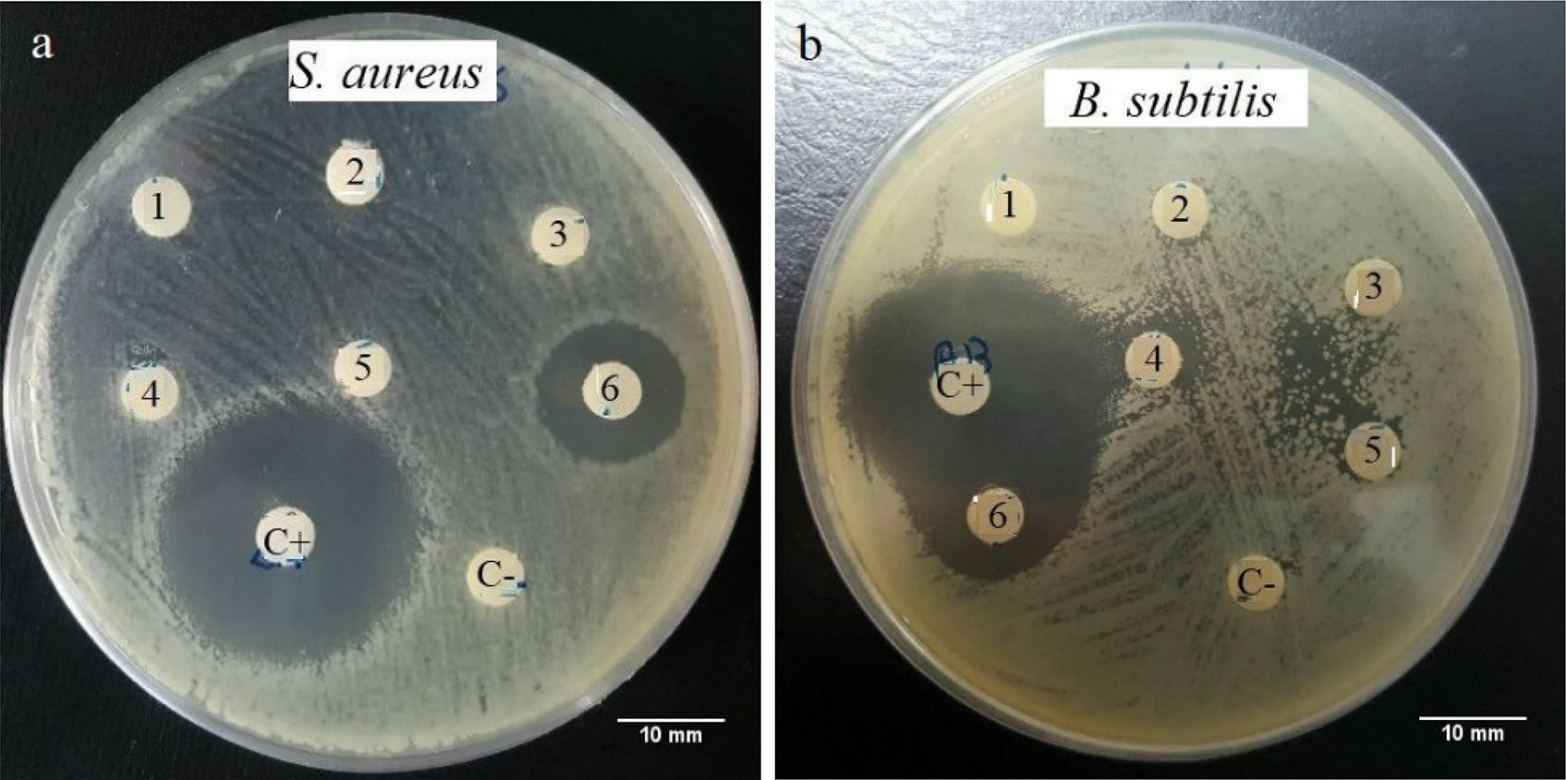
The result of Culture plates depicts radial diffusion assay (RDA) for the antibacterial activity of the HPLC peaks 1–6 from < 5-kDa, and blank sterile Antibiotic disk (C+) and blank sterile disk (C−) against *S. aureus* (**a**) and *B. subtilis* (**b**).

Peaks 1–6 were individually collected and assayed for antimicrobial activity. Fraction P6 (corresponding to 65–75% ACN) showed the activity against *S. aureus* and *B. subtilis* among all fractions attained (Table 3, Fig. 4). Then it was sub-fractionated into four additional peaks (P6-1, P6-2, P6-3, P6-4) when analyzed by the same RP-HPLC with an ACN shallow gradient (50–80%) (Fig. 3). Antimicrobial activity was assessed for each peak, and P6-2 represented antibacterial activity.

**Table 3:** Protein content and antibacterial activity of the HPLC peaks 1–6 from < 5 kDa and blank sterile Antibiotic disk (C +) against *S. aureus* and *B. subtilis*0 (n = 3).

Samples	Protein concentration (µg/ml)	Inhibition diameter (mm)		Percent value (%)	
		*S. aureus*	*B. subtilis*	*S. aureus*	*B. subtilis*
P1	nd	nd	nd	nd	nd
P2	nd	nd	nd	nd	nd
P3	nd	nd	nd	nd	nd
P4	nd	nd	nd	nd	nd
P5	nd	nd	nd	nd	nd
P6	600	17.5 ± 1.5^a^	21 ± 2^a^	55.55 ± 1.5^a^	63.63 ± 2^a^
C + (AB)	–	31.5 ± 1^b^	33 ± 1^b^	100^b^	100^b^

Different letters indicate significant differences (*p* < 0.05). nd: Not detected.

### Antioxidant activity

**Figure 5:**
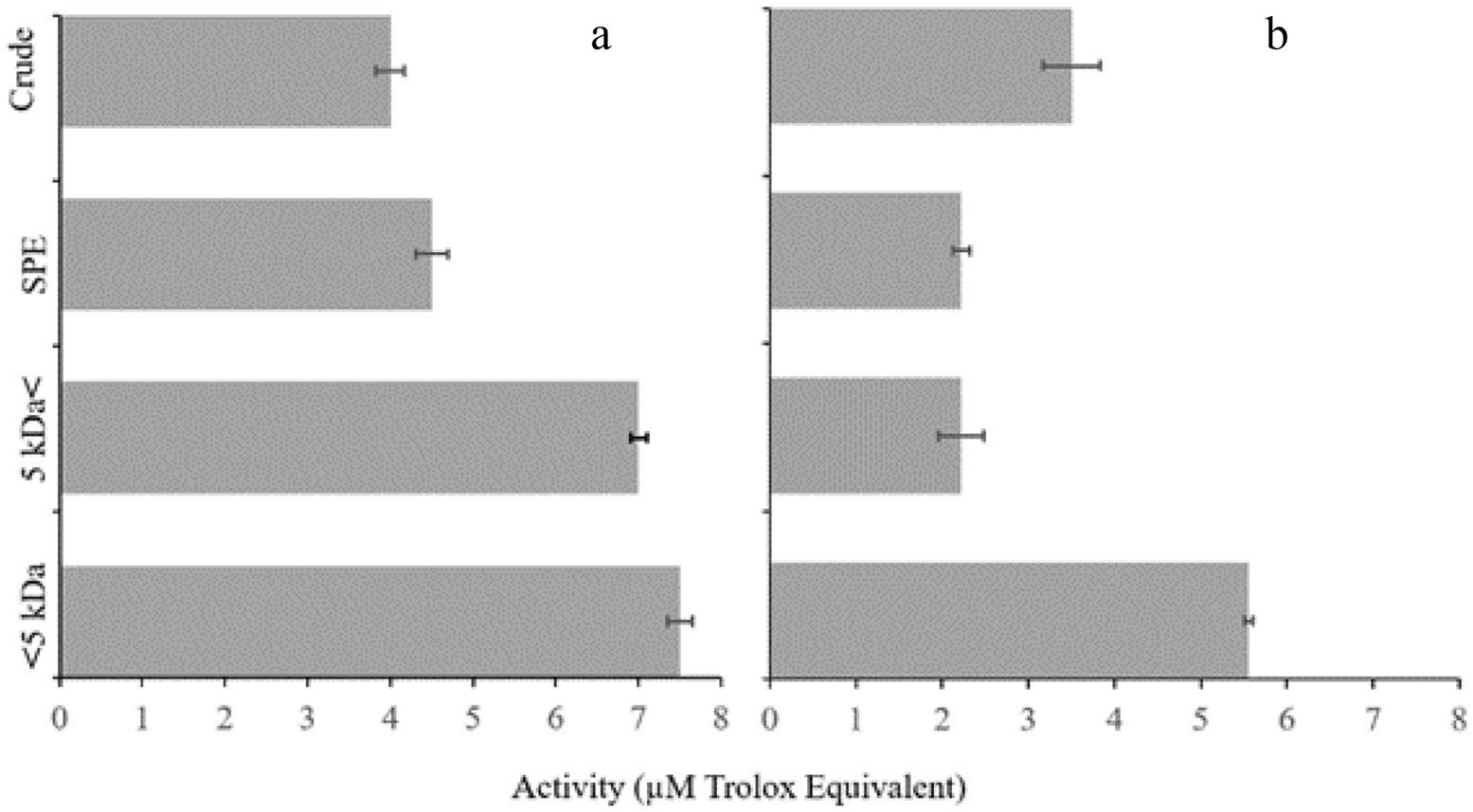
Free radical scavenging activities of the crude epidermal mucus, SPE elutes, and the fractions of 5 kDa < and < 5 kDa against ABTS (**a**) and DPPH (**b**) radicals. Values represent the mean ± SD of three repeats.

The antioxidant activities of the epidermal exudates, SPE elutes, and ultrafiltration-derived fractions were determined using DPPH and ABTS (µM Trolox equivalent). Table 4 indicated that < 5 kDa fraction exhibited the highest scavenging activity against ABTS and DPPH free radicals as 7.5 and 5.55 µM Trolox E, respectively. Furthermore, compared to the DPPH method, all samples showed higher radical ‏scavenging capacity in the ABTS (Fig. 5 and Table 4).

**Table 4:** The total protein contents and antioxidant activity of the epidermal exudates' and the size-based fractionations of *N. fluviatilis pallasi.*

Samples	Protein concentration (mg/ml)	Antioxidant activity TAEC (µM Trolox E)	
		ABTS	DPPH
Crude	0.4^a^	4 ± 0.17^a^	3.5 ± 0.22^a^
SPE	4 ± 0.25^b^	4.5 ± 0.22^a^	2.22 ± 0.3^a^
5 ˂ kDa	7.5 ± 0.42^c^	7 ± 0.11^b^	2.22 ± .012^a^
5 ˃ kDa	4.2 ± 0.22^b^	7.5 ± 0.17^b^	5.55 ± 0.33^b^

Different letters indicate significant differences (*p* < 0.05).

## Discussion

Nowadays, natural products are preferred in foods, feeds, and therapeutics as functional foods, nutraceuticals, or additives instead of chemicals and synthetics. Among them, BAPs have attracted much attention. Although they have been isolated from various sources, the fish protein hydrolysates (FPH) are the most reported when fish is considered the source^12^. Fish skin mucus has recently been recognized as a potential BAP source, providing the first line of defense against invading pathogens. Here it was reported the skin exudates-isolated BAPs of Caspian sand goby, *N. fluviatilis pallasi,* as hydrolysis-independent bioactive molecules.

The antimicrobial activity of crude epidermal mucus was not detected by radial diffusion, probably due to the high viscosity of the mucus or low concentration of components with antimicrobial activity^39^. Similar results about Atlantic Halibut^39^, two species of stingray (*Dasyatis sephen* and *Himantura gerrardi*)^50^, the climbing perch, *Anabas testudineus*^51^ have also been reported. Once the mucus had been homogenized in the extraction medium, centrifuged, and concentrated by SPE on a Sep-Pak C18 column, the activity was detected for the first time.

Protein samples usually contain substances that interfere with used downstream methods. There are several strategies to remove unwanted materials from the samples. One of the most appropriate approaches is protein precipitation. Compared to dialysis or gel filtration methods (Desalination columns), this method allows protein samples to concentrate and purify from undesirable materials simultaneously. However, a disadvantage of this approach is that proteins may be denatured. On the other hand, there is a concern about the loss of some proteins during the precipitation stages. Many studies successfully applied these goals achievement with SPE method^26,30,36,39–41,43,44,52^.

Ultrafiltration is a quick and straightforward convective process for isolating molecules, primarily on a molecular weight basis, without the need for multiple washing times and prolonged separation stages to obtain low MW compounds (i.e., < 1 kDa fractions)^53^. Compared to other alternative procedures such as gel chromatography, membrane technology is a gentle, non-denaturing approach that presents multiple benefits such as high products throughput capacity and purity under ambient conditions, lower capital investment, ease of conversion to large-scale commercial production, higher output, and easy-cleaning supplies^53^.

The protein content of different fractions showed that significant amounts of protein are missed in the fractionation process, which is attributed to protein absorption or deposit on the outer (external fouling) or the inside (internal fouling) surface of the membrane of filters^54^. Thus we decided to use a filter (5-kDa). Therefore, antibacterial activity against bacterial species for fractions ˂ and ˃ 5-kDa took place after concentration with SPE and fractionation with filter 5-kDa. The result indicated that low-molecular-weight peptides possess better antibacterial activity than high-molecular-weight peptides, which agrees with former findings^26,30,36,38,40,43^. Many studies showed that low-molecular-weight peptides represent a better interaction with bacterial membranes as the membrane interaction is an essential determinant for the direct antimicrobial action of AMPs, both whenever the membrane itself is a target and whenever intracellular targets need to be accessed through translocation^55,56^. Also, peptides with small sizes (˂10 kDa) disturb the structure of lipid bilayers easier and make cytoplasmic content leak with subsequent cell deaths^57^.

Electrostatic attractions between the cationic AMPs and bacterial surface with the negative charge are essential factors for the adsorption of AMPs on bacteria^58,59^. The outer cytoplasmic membranes of both Gram-negative and Gram-positive species are made up of chiefly phospholipids with negatively charged head groups such as phospholipids phosphatidylserine (PS), phosphatidylglycerol (PG), cardiolipin and highly attractive for positively-charged AMPs^59^. The existence of teichoic acids in the Gram-positive bacterial cell wall and LPS in the Gram-negative bacterial outer membrane each exhibit an extra electronegative charge to the surface of bacteria^58^. Moreover, compared with Gram-negative bacteria, Gram-positive species possess a higher percentage of negative charge PG comprising saturated and unsaturated fatty acids in their cell membrane^58^. For example, *Clostridium difficile*, *Streptococcus sanguis*, *B.subtilis* and *S. aureus* have 100, 82, 70, and 43% PG respectively. Besides, gram-positive species with cocci form are loaded with PG, while bacilli have a vast quantity of phosphatidylethanolamine^58^.

In the human body, extreme free radicals can damage and age. Antioxidant agents can decrease the number of free radicals in the body and protect humans from various disorders. Hence, the research for finding antioxidants, particularly new antioxidant agents with natural origins, has received more and more attention. Fish skin and its secretions include multiple peptides with biological activities, which may be utilized in the imminent future as therapeutic agents. Nowadays, investigation of skin-isolated antioxidant peptides from various species revealed that antioxidant and antimicrobial peptides possessed the common origin^60^, showing a close evolutionary relation. Therefore, in this study, we also studied the antioxidant activity of the epidermal exudates and the size-based fractionations of *N. fluviatilis pallasi* for the first time.

Various techniques have been reported to assess the total antioxidant capacity of food and animal tissues. The FRAP, DPPH, and FRAP tests are the most typical methods commonly applied in numerous investigations. Each of the reported methods shows a particular mechanism of action with advantages and disadvantages; nonetheless, in the absence of a comprehensive method that can lead to precise data on a given sample, the best strategy is to adopt several methods simultaneously. Based on Zou et al.^61^, owing to the various basic mechanisms between investigation assays, the values or variation trends taken via diverse assays are varied widely, even concerning the identical amino acid residues and composition, whereas all these assays represent a single feature. Therefore, in this study, standard curves based on different concentrations of Trolox were made for each test, and the obtained data from each test were reported as µM Trolox E unit. In this way, it was possible to rank and compare the data without considering the method of measuring antioxidant properties.

Several studies have examined the contribution of molecular size and structural properties of peptide mixtures in protein hydrolysates to their bioactivity. These studies showed that peptide fractions with low molecular weight generally are more potent antioxidant agents^19,21,62–64^, as these peptides exhibit a better chance to pass the intestinal barrier and to fulfill biological action. Moreover, it has been reported that low-molecular-weight fractions have more donating electron/hydrogen peptides that can efficiently react with free radicals and produce stable products, stop chain elongation as well as inhibit the lipid peroxidation cycle spread^22,65^. Therefore, they exhibit higher antioxidant activity.

The cationic properties of the amino acid composition, high levels of total hydrophobic have been proposed to be the crucial factors governing antioxidant activity^62^. The hydrophobic peptides show the potential to communicate with lipid molecules and scavenge by donating protons to lipid-derived radicals^63^. On the other hand, the accessibility of hydrophobic antioxidative peptides to hydrophobic cellular components could be increased, thereby promoting communication with radical species^66^. Moreover, some amino acids play a significant role in the peptide radical scavenging capacity because of their specific structural features.

It was also observed that all samples showed higher radical ‏scavenging capacity in the ABTS method than in the DPPH method. The same reports were found in other antioxidative compounds^67^, including peptides^60^ that several peptides only possessed ABTS‏ scavenging action, however, they did not represent DPPH free radical scavenging action. Nevertheless, the precise reason has not been cleared yet. A logical description is that the group engaged in scavenging DPPH free radicals in antioxidative compounds is influenced by spatial (steric) hindrance that arose from its molecular structure, failing in scavenging free radicals^68^. The molecular structure of ABTS compared with the DPPH may facilitate the oxidant functional groups' access to these peptides.

## Conclusion

As WHO has declared that multi-drug resistant is one of the top 10 global public health threats against humanity, the rising concerns on its development caused finding potential therapeutic molecules that are effective both facing infectious and non-infectious diseases. In this regard, Bioactive peptides opened a novel avenue as alternative pharmaceutical drugs owing to their widespread applications. The presented study for the first time investigated skin mucus Caspian sand goby, as a fundamental non-specific immune component, for discovering the peptide agents. The results show that the epidermal exudates and their size-based fractionations represent in vitro antimicrobial and antioxidant properties. These capacities were found to be higher, mainly in the low-molecular-weight fractions. Besides, the increase in antibacterial and antioxidant capacities might be associated with the higher content of cationic and hydrophobic amino acids in fractions. This study demonstrates that the skin mucus of Caspian sand goby may serve as a good source of desirable bioactive peptides for playing an excellent role in the developing aquaculture and human health-related practical applications.

### Supplementary Information

Supplementary Information.

## Data Availability

The data presented in this study are available in the article and Supplementary Information file.
