# Supplementary material for: Fractionation of the Caspian sand goby epidermal exudates using membrane ultrafiltration and reversed-phase chromatography: an investigation on bioactivities
Source: Sci Rep. 2024 Jan 19;14:1716. doi: 10.1038/s41598-024-52126-z (PMC10799039; doi:10.1038/s41598-024-52126-z)

Fractionation of the Caspian sand goby epidermal exudates Using Membrane Ultrafiltration and Reversed-phase chromatography: an investigation on bioactivities

Mohammad Akhavan-Bahabadi, Hamed Paknejad, Aliakbar Hedayati , Mehran Habibi-Rezaei

Supplementary Information

Supplementary Figure S1

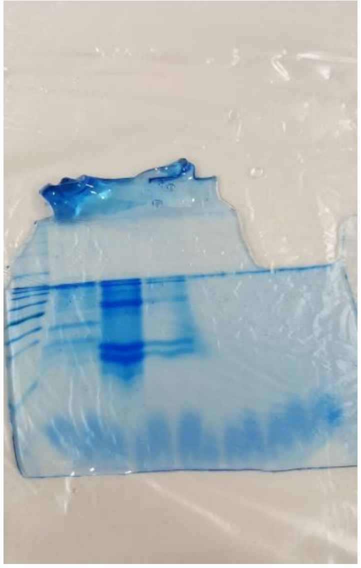

Fig 1.2. The full-length (uncropped) SDS-PAGE of the samples

Supplementary Figure S2

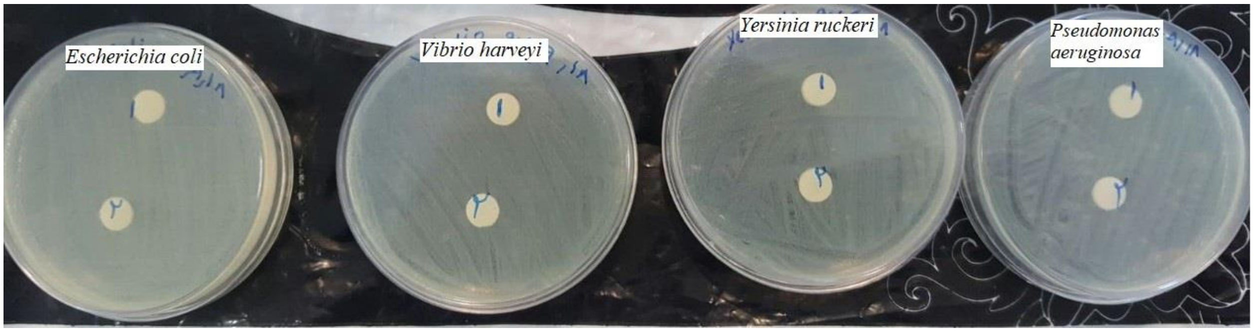

The result of Culture plates depicts radial diffusion assay (RDA) for the antibacterial activity of the fractions <5 kDa, 5 kDa< against the Gram-negative bacteria used in this study, including *Escherichia coli*, *Pseudomonas aeruginosa*, *Vibrio harveyi* and *Yersinia ruckeri.*

Supplemental Tables

Supplementary Table S1

Different concentrations (mg) used for 5< and 5> KDa fractions against *S.aureus* and *B.subtilis* and their absorbance at 600 nm for determining the MIC values (The lowest concentration of an antimicrobial that will inhibit the visible growth of a microorganism after overnight incubation)

	**1**	**2**	**3**	**4**	**5**	**6**	**7**	**8**	**9**	**10**	**11***	**12****
Various con. 5<	3.75	1.875	0.937	0.468	0.234	0.117	0.058	0.029	0.014	0.007	11	12
**A**: *B.subtilis,* 5<	0.132	0.059	0.327	0.318	0.320	0.292	0.319	0.320	0.336	0.597	0.607	0.347
**B**: *B.subtilis,* 5<	0.069	0.34	0.052	0.323	0.320	0.315	0.315	0.320	0.351	0.926	0.94	0.348
**C**: *S. aureus,* 5<	0.375	0.349	0.321	0.317	0.324	0.317	0.326	0.312	0.373	0.715	0.886	0.346
**D**: *S. aureus,* 5<	0.379	0.36	0.325	0.325	0.316	0.314	0.315	0.313	0.374	0.893	0.821	0.344
Various con. 5 >	2.236	1.118	0.559	0.279	0.139	0.069	0.034	0.017	0.008	0.004	11*	12**
**E**: *S. aureus,* 5>	0.121	0.333	0.344	0.324	0.32	0.32	0.316	0.312	0.322	0.349	0.888	0.343
**F**: *S. aureus,* 5>	0.067	0.308	0.317	0.321	0.321	0.307	0.312	0.313	0.325	0.689	0.805	0.344
**G**: *B.subtilis,* 5>	0.046	0.049	0.348	0.316	0.282	0.311	0.309	0.312	0.315	0.562	0.692	0.339
**H**: *B.subtilis,* 5>	0.047	0.043	0.308	0.319	0.314	0.315	0.305	0.31	0.314	0.548	0.723	0.336

*11: bacteria + culture media (negative control), **12: culture media (positive control)

Supplementary Figure S3

Different concentrations (mg) used for 5< and 5> KDa fractions against *S.aureus* and *B.subtilis* and their absorbance at 600 nm for determining the **MIC values** (The lowest concentration of an antimicrobial that will inhibit the visible growth of a microorganism after overnight incubation)

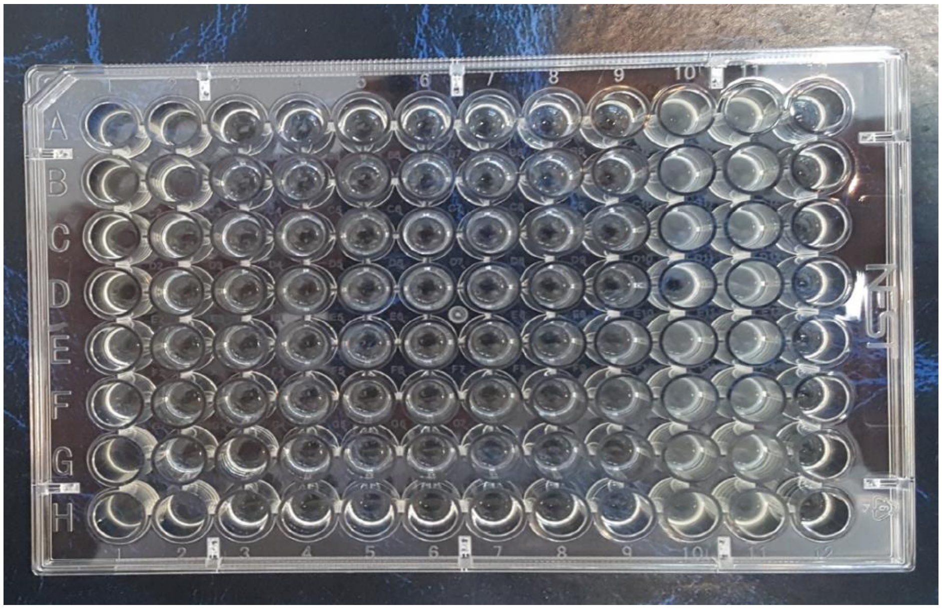

MIC assay of different fractions against selected bacteria

Supplementary Figure S4

MBC assay of different fractions against selected bacteria (The lowest concentration of an antimicrobial that will prevent the growth of a microorganism after subculture on to antibiotic-free media)

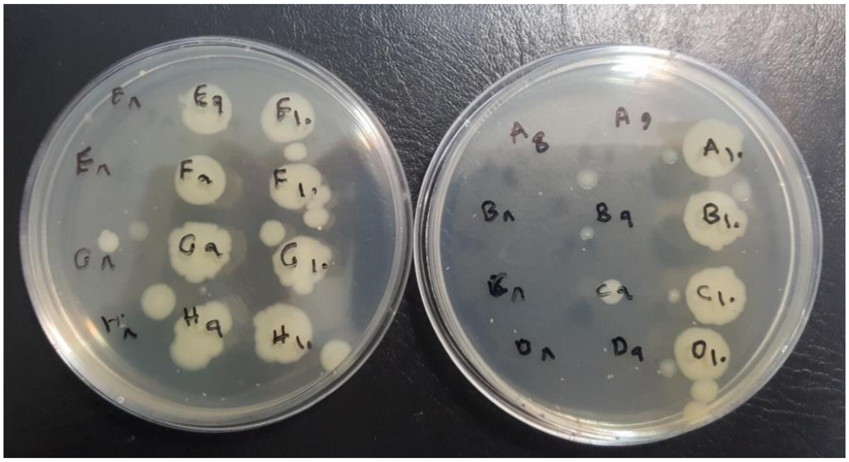

Supplement: Supplementary file 1 — Supplementary Information. [file 41598_2024_52126_MOESM1_ESM.pdf]
